# Early cessation of pressure garment therapy results in scar contraction and thickening

**DOI:** 10.1371/journal.pone.0197558

**Published:** 2018-06-13

**Authors:** Danielle M. DeBruler, Jacob C. Zbinden, Molly E. Baumann, Britani N. Blackstone, Megan M. Malara, J. Kevin Bailey, Dorothy M. Supp, Heather M. Powell

**Affiliations:** 1 Department of Materials Science and Engineering, The Ohio State University, Columbus, OH, United States of America; 2 Department of Biomedical Engineering, The Ohio State University, Columbus, OH, United States of America; 3 Department of Surgery and Division of Critical Care, Trauma and Burns, The Ohio State University, Columbus, OH, United States of America; 4 Research Department, Shriners Hospitals for Children, Cincinnati, OH, United States of America; 5 Department of Surgery, University of Cincinnati, Cincinnati, OH, United States of America; Medical University of Graz, AUSTRIA

## Abstract

Pressure garment therapy is often prescribed to improve scar properties following full-thickness burn injuries. Pressure garment therapy is generally recommended for long periods of time following injury (1–2 years), though it is plagued by extremely low patient compliance. The goal of this study was to examine the effects of early cessation of pressure garment therapy on scar properties. Full-thickness burn injuries were created along the dorsum of red Duroc pigs. The burn eschar was excised and wound sites autografted with split-thickness skin. Scars were treated with pressure garments within 1 week of injury and pressure was maintained for either 29 weeks (continuous pressure) or for 17 weeks followed by cessation of pressure for an additional 12 weeks (pressure released); scars receiving no treatment served as controls. Scars that underwent pressure garment therapy were significantly smoother and less contracted with decreased scar height compared to control scars at 17 weeks. These benefits were maintained in the continuous pressure group until week 29. In the pressure released group, grafts significantly contracted and became more raised, harder and rougher after the therapy was discontinued. Pressure cessation also resulted in large changes in collagen fiber orientation and increases in collagen fiber thickness. The results suggest that pressure garment therapy effectively improves scar properties following severe burn injury; however, early cessation of the therapy results in substantial loss of these improvements.

## Introduction

Pressure garment therapy (PGT) has been used to reduce scarring following burn injuries, resulting in improvements in erythema, scar height, and scar pliability[1–4]. Unfortunately, a major obstacle of PGT is low patient compliance. A survey of 100 adult pressure garment users found that less than half were in complete compliance with the therapy[5]. Another study conducted on the use of tubular compression bandages found an average of 30% of the study population reported poor compliance. In addition to observing poor compliance within the study population, outcomes were correlated with patient compliance. Patients who were compliant had a higher probability of a “good” clinical outcome (58%), whereas non-complaint patients had a substantially lower probability of exhibiting good clinical outcomes (16%). [6]. Therefore, therapeutic outcomes could be improved by increasing patient compliance.

On average, patients are instructed to wear the pressure garments for 1 year following burn injury [2][7], though the recommended time frame varies widely from as little as 6 months[1,8] to as long as 2 years[9,10]. An early study that used adhesive sponges and elastic bandages to apply pressure following burn injury suggested that the therapy was effective even when discontinued after only 4 months[11]. The recommended time frame for PGT is often based on the physician’s experience, and there has never been a controlled experimental study to suggest the necessary length of time that pressure garments should be worn, or to examine the effects of early removal. Because the garments are reported to be uncomfortably hot and itchy[12], patients may be more likely to wear them if the therapy could be discontinued after a shorter period of time.

The goal of this study was to observe the effects of removing pressure garments four months after therapy was initiated. Red Duroc pigs that had been undergoing PGT for 17 weeks after burn injury were used in the experiment. Half of the wounds continued PGT for an additional 12 weeks, while the other half was released from pressure. Scar characteristics including scar area, roughness, depth, and scar biomechanics were assessed and tracked over time.

## Materials and methods

### Animal care and scar formation

All experiments were conducted using female red Duroc pigs following a protocol approved by the Ohio State University Institutional Care and Use of Animals Committee (Protocol #2014A00000072). Anesthesia was initiated with Telazol (Zoetis, Florham Park, NJ) and maintained with isoflurane. Following shaving, alternating scrubs of 2% chlorohexidine and 70% isopropyl alcohol (Butler Schein, Columbus, OH) were used to clean the dorsal trunk. Prior to wounding, a 1x1 cm grid was tattooed on each side of the pig to track skin surface area increase with time. Thermal injuries were utilized to create full-thickness, cutaneous defects as scarring in porcine skin was previously reported to be exacerbated in burn injuries and to more closely mimic human hypertrophic scar compared to scars resulting from excisional injuries [13]. To create full-thickness burn wounds, a 1” x 1” custom metal stylus[14] was heated to 200°C and held against each side of the pig (N = 4 pigs) for 40 seconds. The burn eschar was excised and the wound bed grafted with split-thickness skin that was harvested from the dorsum using a Zimmer Air Dermatome (Zimmer, Warsaw, IN) and meshed 1.5:1. Saline soaked surgical sponges (Hydrasorb®, Carwild Inc. New London, CT) were packed into the wounds and secured with sterile spandex and skin staples (Henry Schein, Melville, NY). A fiberglass cast (3M Helathcare, St. Paul, MN) was formed over the back of the pig to protect the healing wounds. The cast was held in place with Vetrap^TM^ (3M Healthcare) and Elastikon® (Johnson & Johnson, New Brunswick, NJ). NOVAPLUS Fentanyl patches (Watson Pharmaceuticals, INC, Parsippany, NJ) were applied for pain management and removed 3 days post wounding. One week following grafting, bandages and bolsters were removed.

### Pressure application and removal

Pressure was applied circumferentially around the dorsum using custom, adjustable pressure garments (Powernet; Darlington Fabrics, New York, NY) fabricated with Velcro™ to allow for pressure adjustments. Powernet fabric was selected as it was previously shown to exhibit lower amounts of fatigue and higher levels of applied pressure for a given reduction in circumference than Dri-Tek Tricot fabrics[15]. Pressure was initiated within one week of grafting and was maintained over the wounds at 20 mm Hg. Polyurethane foam was used to ensure even pressure over concave areas, and pressure was checked using a Kikuhime pressure sensor (MediGROUP, Melbourne, Australia). Pressure beneath the garments and directly over the scars was checked once per day and adjusted if not at the targeted 20 mm Hg. Every three days, garments were removed and replaced with freshly laundered garments. 17 weeks after the initial wound creation, pressure was released from half of the pressure treated wounds and maintained in the other half for an additional 12 weeks, resulting in three treatment groups: controls (no pressure applied throughout the experiment), continuous pressure group (pressure applied within 1 week of grafting and maintained for a total of 29 weeks) and a pressure released group (pressure applied within 1 week of grafting for 17 weeks, then removed for the final 12 weeks). Each pig contained both treatment groups and controls with sites randomized (N = 8 scars per group).

### Scar area

Immediately after burn excision and at 1, 3, 5, 11, 17, 21, 25 and 29 weeks after grafting, the boundaries of the scars (n = 8 per group per time point) were traced using a transparent film. The tracings were scanned with a ruler in the field of view, imported into ImageJ, and the scar area was quantified. The area at each time point was normalized to the area of that scar at the initial time point (week 0, post excision) to obtain a percent area for each scar. Average % area ± standard error of the mean (SEM) is reported. Growth of the pigs was calculated by tracking the increase in surface area using the tattooed grid. The area of six, initially 1 by 1 cm squares was measured on each pig at each time point, normalized to the area of that square at week 0, and averaged to obtain a percent increase in surface area due to the growth of the pig.

### Scar roughness

Surface roughness of the scars was quantified using ten-point mean roughness, or Rz. Aquasil Ultra XLV dental impression material (DENTSPLY Caulk, Milford, DE) was applied to the scars to create a negative impression (or mold) of the surface topography of the scars. Molds from weeks 17 and 29 (n = 8 per group) were filled with Phase2Gel dental alginate (Accu-Cast Dental, Bend, OR) to create a positive impression of the surface of the scars. The alginate was removed and cut into slices. The cross sections of the slices were imaged with a ruler in the field of view, and the pictures were imported into ImageJ. Rz was obtained by measuring the depth of the 5 largest peaks in each tracing and averaging them. Three representative cross sections were used and averaged for each scar. Average Rz ± SEM is reported.

### Scar height

Molds from weeks 17 and 29 were also used to measure the volume of the scars (n = 8 per group except Continuous Pressure group at week 29 where mold was too warped to collect reliable volume measurement). The negative molds were filled with Phase2Gel dental alginate until they were flush with the level of surrounding uninjured skin. The molds were weighed before and after being filled with alginate. The difference in the weight was used to calculate the volume by using the density of the dental alginate. The average height of the scars above uninjured skin was then calculated by dividing the volume of the scar by the scar area at that time point. For scars that were below the level of the surrounding normal skin, a positive replica of the mold was created. The mold was circled with fast dry acrylic latex caulk (DAP Products, Inc, Baltimore, MD) to include both the scar and the surrounding normal skin. After the caulk dried, casting resin (Alumilite Corp. Kalamazoo, MI) was poured into the mold and allowed to harden. The resulting positive resin mold was then weighed, filled with alginate, and weighed again to obtain the negative volume of skin below the surface of the normal skin. Dividing this volume by the area of the scar at that time point resulted in how far the surface of the scar was below the surrounding normal skin. These distances are reported as average scar height from normal skin ± SEM.

### Scar biomechanics

At week 17 post-injury (prior to removal of PGT in the release group) and at the final time point (week 29), *in vivo* biomechanics were assessed using a torsional ballistometer (Dia-Stron, Inc.; Clarksburg, NJ). The ballistometer drops a small probe onto the skin from a known distance, k, and measures the rebound bouncing of the probe until it comes to rest. The initial penetration of the probe into the skin, or the indent (mm), can be used as a measure of the hardness of the skin. Softer, more pliable skin will result in deeper penetration of the probe, and a greater indent. K-values between 1.15 and 1.45 mm were considered reliable thus leaving an n = 8 for all groups at all time points with the exception of the Pressure Released group where n = 6. Average indent ± SEM is reported.

### Scar morphology

**Fig 1:**
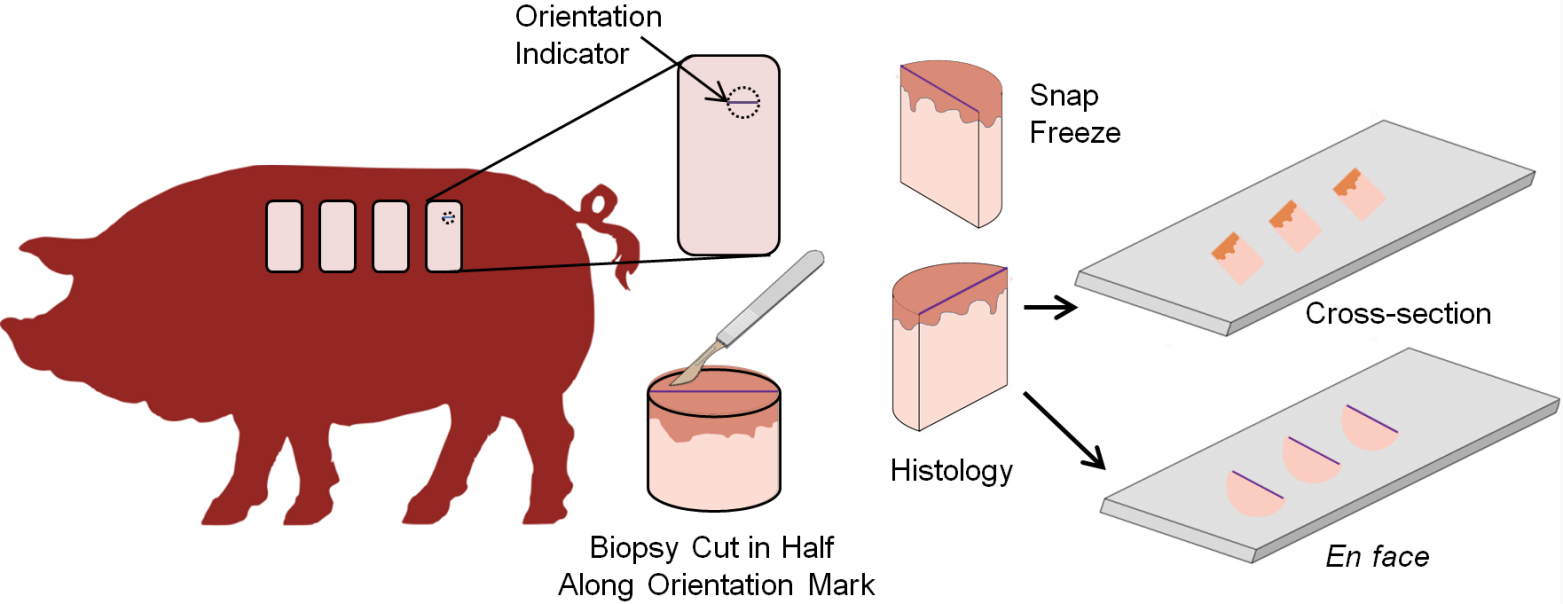
Schematic of the scar biopsy processing methodology. At each time point, at least one 6-mm biopsy punch was collected. Scars were marked to identify tissue orientation with respect to anatomy. The biopsy was cut in half with one half snap frozen and the other half processed for histology. The biopsy was first sectioned in cross-section. Subsequently, the biopsy was sectioned *en face* following removal of the epidermis with coarse sectioning.

At weeks 17, 21, 25 and 29, six mm biopsies were taken of the scars for cryosectioning. For each biopsy, a line was drawn across the scar in the cranial-caudal direction to ensure that all samples were sectioned in the same orientation. Half of the biopsy was snap frozen with the other half prepared for histological analysis. Biopsies were first sectioned in cross-section to assess whole tissue structure. To examine collagen orientation, the epidermis was then removed from the frozen tissue by coarse sectioning, the dermis was sectioned *en face* at 7 μm and sections were stained with hematoxylin and eosin (Fig 1). Images were collected of sections 60–75μm below the dermal-epidermal junction with the dorsal axis of the tissue aligned with the top of each image (n = 6 samples per group per time point). At weeks 17 and 29, additional biopsy punches were collected, fixed in 10% formalin (Sigma-Aldrich, St. Louis, MO) for 72 hours and embedded in paraffin. The samples were sectioned at 10 μm and stained with Masson’s Trichrome (Sigma-Aldrich, St. Louis, MO) and Picrosirius Red (Electron Microscopy Sciences, Hatfield, PA) to visualize scar morphology. A polarized light microscope (Zeiss Axioskop Widefield LM, Oberkochen, Germany) was used to image Picrosirius Red stained sections.

### Statistics

A One-way Analysis of Variance (ANOVA) was used with a Tukey’s post-hoc test (JMP, SAS Institute Inc., Cary, NC). For pairwise comparisons, a student’s t-test was performed. Pig identification was blocked for random effects and statistical significance was considered at p < 0.05.

## Results

### Scar appearance

**Fig 2:**
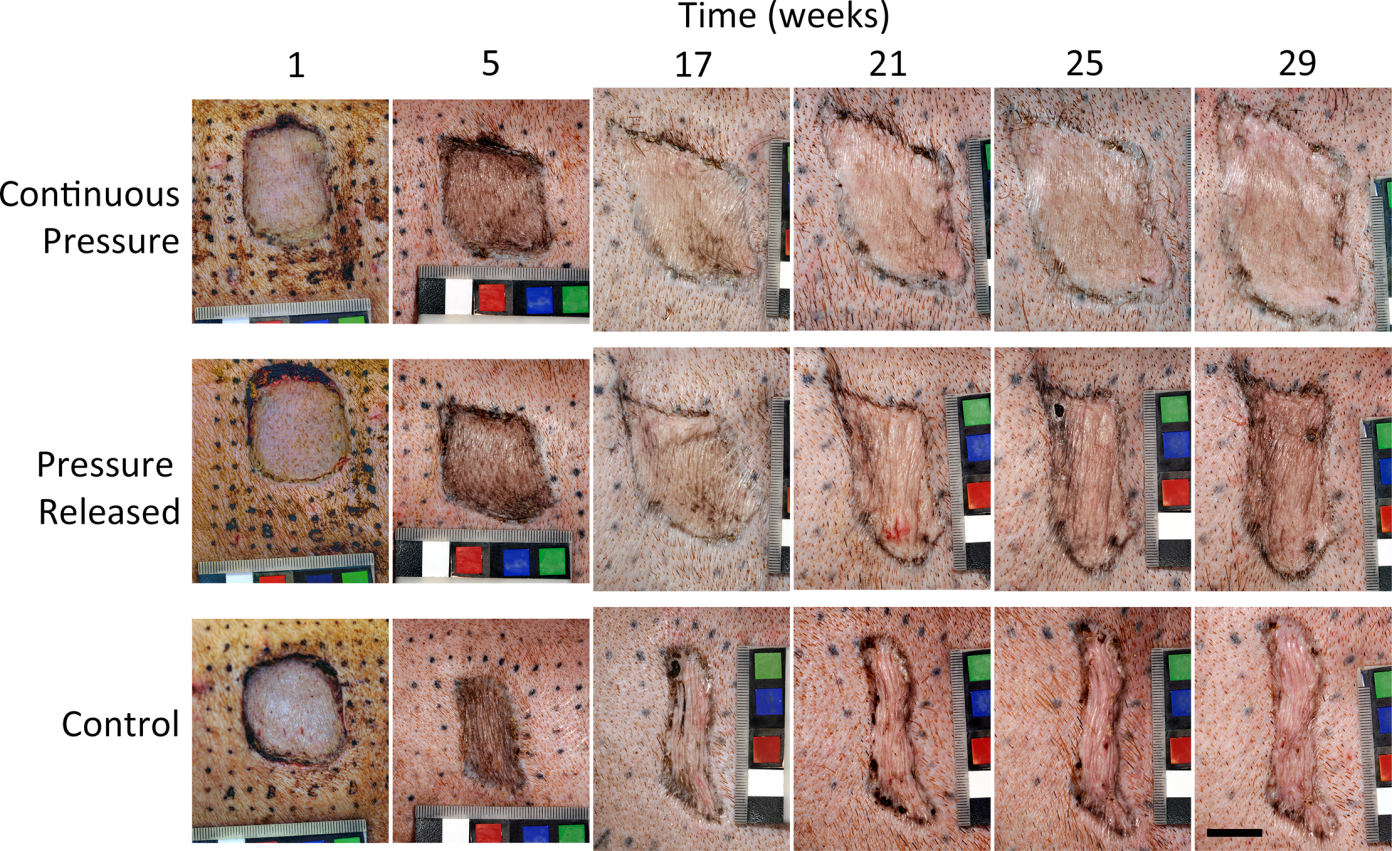
Representative images of scars. Scars were either treated continuously with pressure garment therapy (continuous pressure), or pressure garment therapy was halted at week 17 (pressure released). Control scars did not receive any treatment. Pressure garment therapy (PGT) resulted in smoother, less contracted scars; however, when pressure was removed at 17 weeks scars became visibly contracted within 4 weeks post therapy cessation. Scale bar = 2 cm.

Pressure treatment resulted in scars that were smooth with less contraction compared to control scars that became rough and elongated (Fig 2). When compression was released after week 17, scars became contracted, rough, and raised, similar to the control scars. Scars that received pressure throughout the experiment maintained a smooth appearance, with shapes more similar to the original square wound.

**Fig 3:**
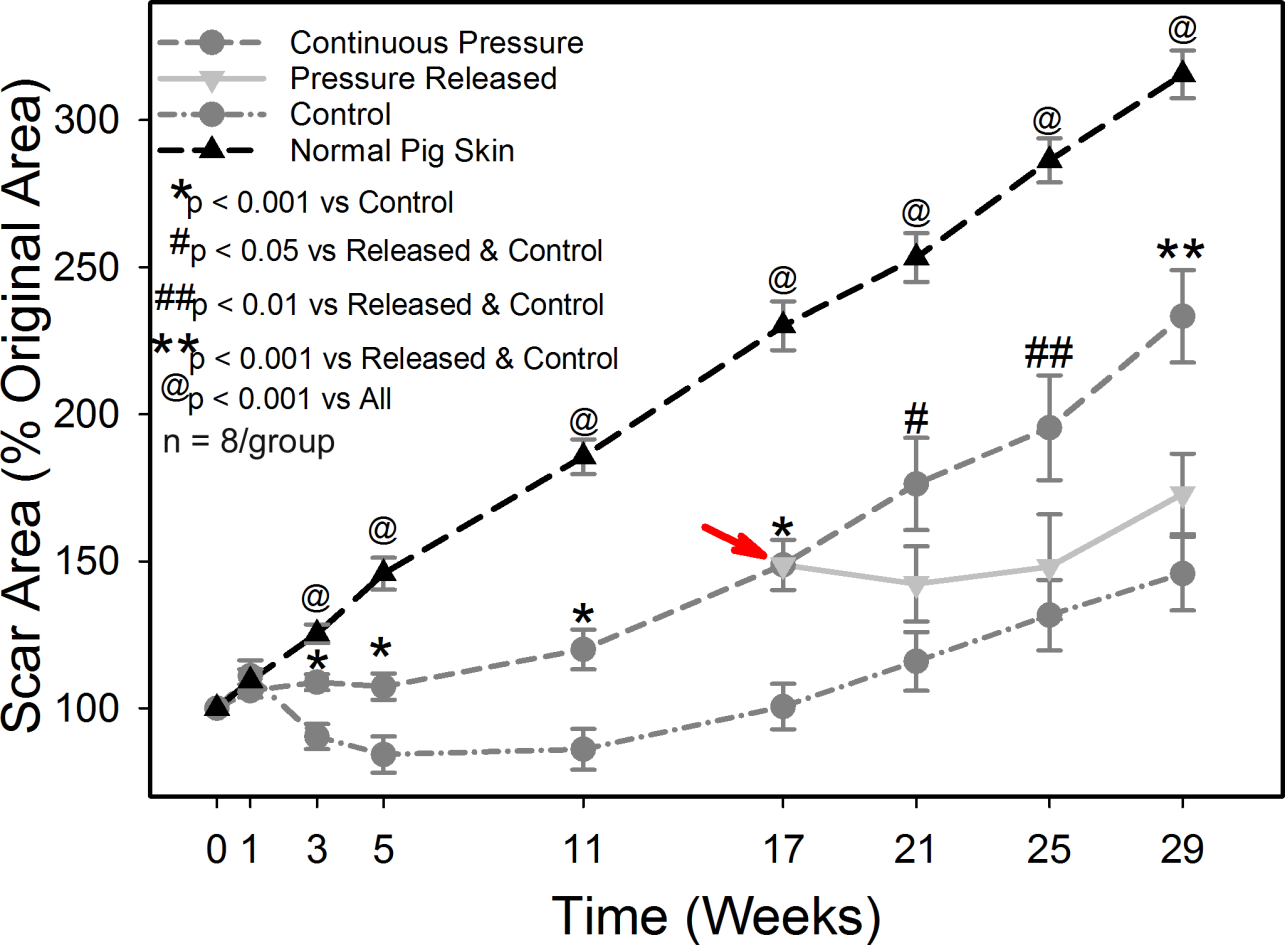
Scar area as a function of time and treatment. Scar area was normalized to the area of each individual scar at week 0 to obtain a percent of the original area. Pressure garment therapy was stopped at week 17 in the pressure released group, indicated by the red arrow. Cessation of PGT resulted in rapid contraction of the skin with scars in the pressure released group exhibiting similar scar areas as untreated controls by week 29 post injury. N = 8 per group.

Scar area was quantified as a measure of the contraction of the scars. From week 3 to week 17, scars that received PGT had significantly greater scar area compared to the control scars but were significantly smaller than normal skin (p < 0.001, Fig 3). By week 17, scars undergoing PGT were an average of 148% of the original wound area, compared to control scars that were an average of 100%. Note that the uninjured skin area measured at week 17 was on average 230% of the original area due to the substantial growth of each animal, thus all scars exhibited significant contraction. When pressure was removed, the scars rapidly contracted, and at four weeks after pressure removal the pressure released group had significantly smaller area than the continuous pressure group, 142% and 176% respectively. For the rest of the study, the continuous pressure group continued to have significantly greater scar area compared to both the pressure released and the control groups (p < 0.05) with no statistical difference in scar area between the control and pressure released groups.

**Fig 4:**
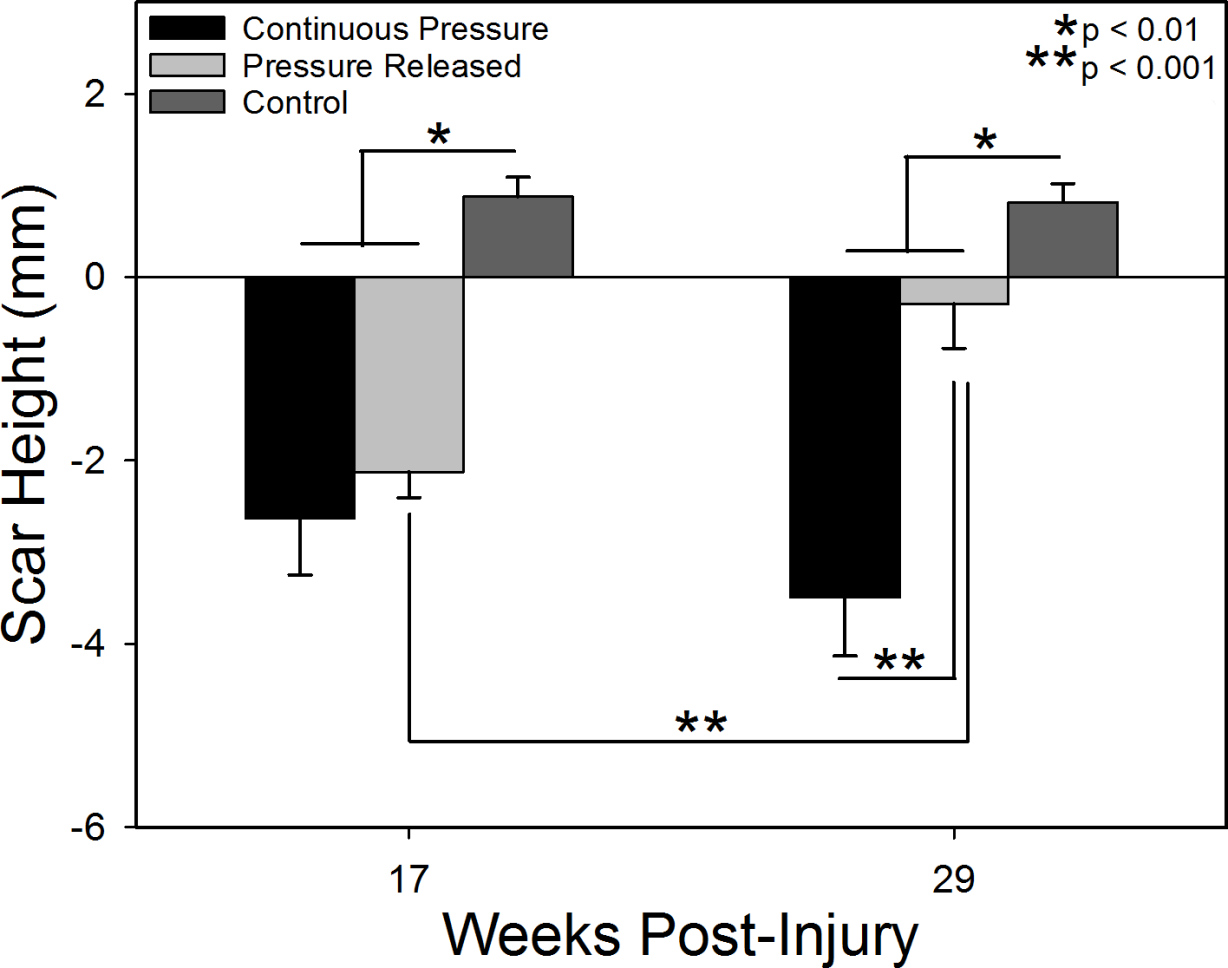
Scar height with respect to the levels of the surrounding normal skin. Negative values represent scars that were depressed with respect to the surrounding tissue and positive values indicate raised scars. Cessation of pressure garment at week 17 resulted in significant scar thickening to raise the scar close to the level of the surrounding tissue. N = 8 per group except N = 7 for Continuous Pressure at week 17.

**Fig 5:**
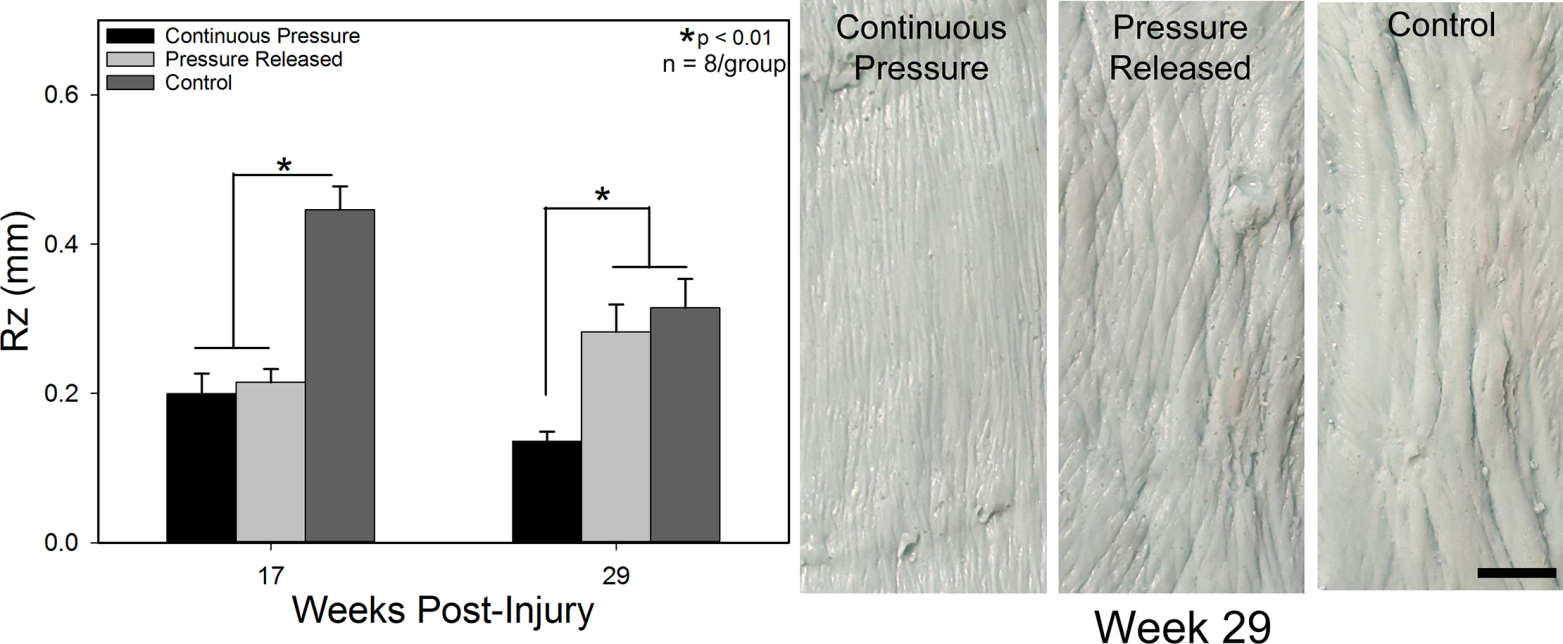
Scar roughness (Rz) as a function of time and treatment. Groups which received PGT were significantly smoother than controls at week 17. Cessation of PGT resulting in an increase in surface roughness versus the continuous pressure group. Photographs of scar replicas at week 29 (*Right)* illustrating the differences in surface texture. Scars treated with continuous PGT were comprised of very fine wrinkles whereas the released and control groups had much larger scale roughness. All images at the same magnification. Scale bar = 1 cm. N = 8 per group.

Scars treated with pressure had significantly reduced scar height and Rz (surface roughness) compared to control scars (p < 0.01; Figs 4 and 5). Scars receiving PGT tended to be below the level of surrounding normal skin and were smooth, while control scars were raised ~1 mm above normal skin and were rougher. After pressure was removed from the pressure released group, the scars increased in height (p < 0.001) and became rougher, similar to the control group, while the continuous pressure group remained smooth with reduced height.

### Biomechanics

**Fig 6:**
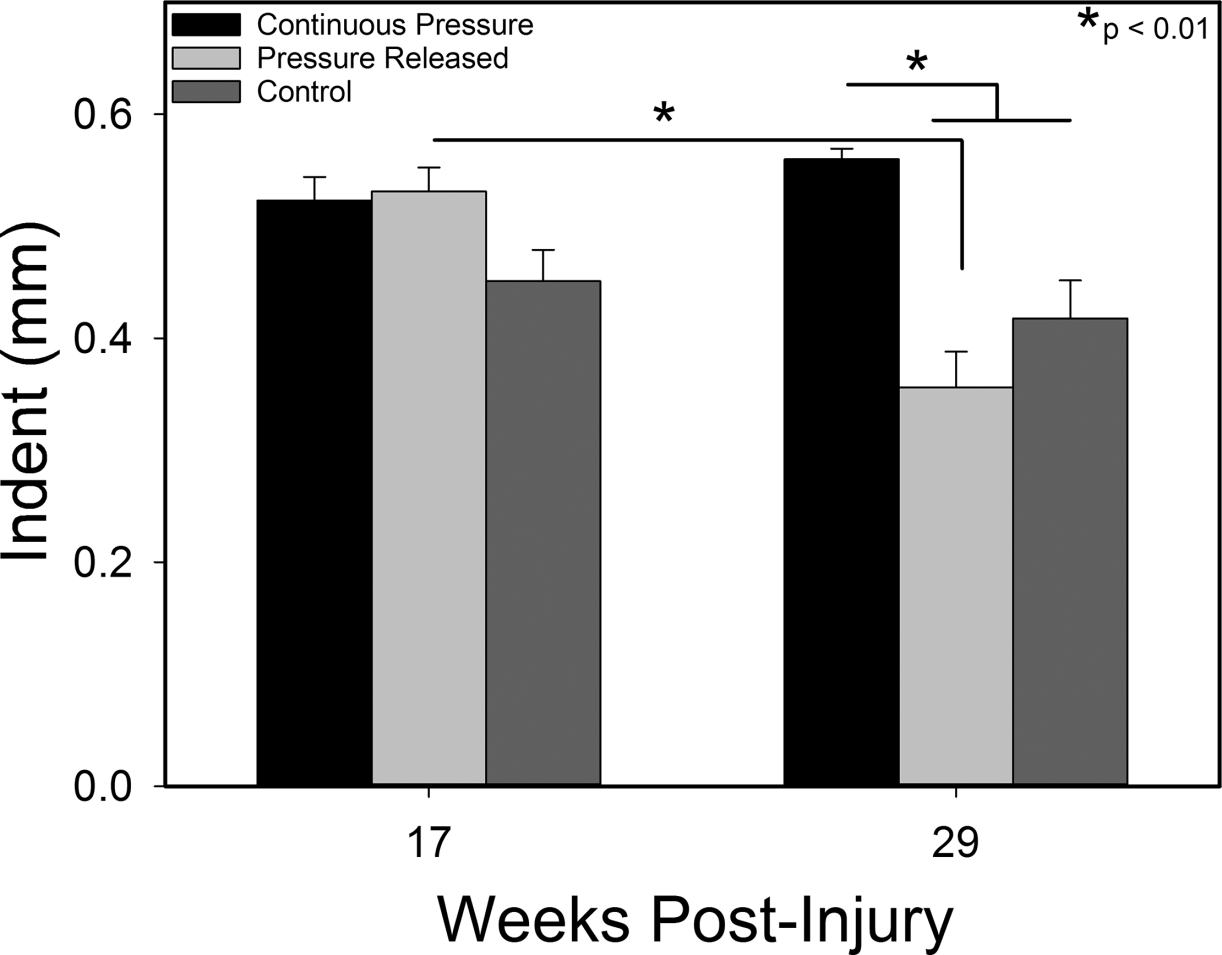
Scar biomechanics as measured with a torsional ballistometer. Deeper indentations (larger values) represent softer, more pliable skin. Pressure garment therapy decreased scar hardness compared to controls. PGT cessation resulted in an increase in hardness of the scars. N = 8 per group except N = 6 Pressure Released group at week 29.

Compression treatment resulted in scars that were more pliable on average, indicated by an increase in the indentation of the ballistometer probe compared to control scars (Fig 6). After pressure was removed, the pliability of the pressure released group decreased significantly (p < 0.01), while the pliability of the continuous pressure and control groups remained relatively constant. By the final time point, the continuous pressure group had a significantly greater indent, and thus greater pliability, compared to the pressure released and control groups (p < 0.01).

### Scar morphology

**Fig 7:**
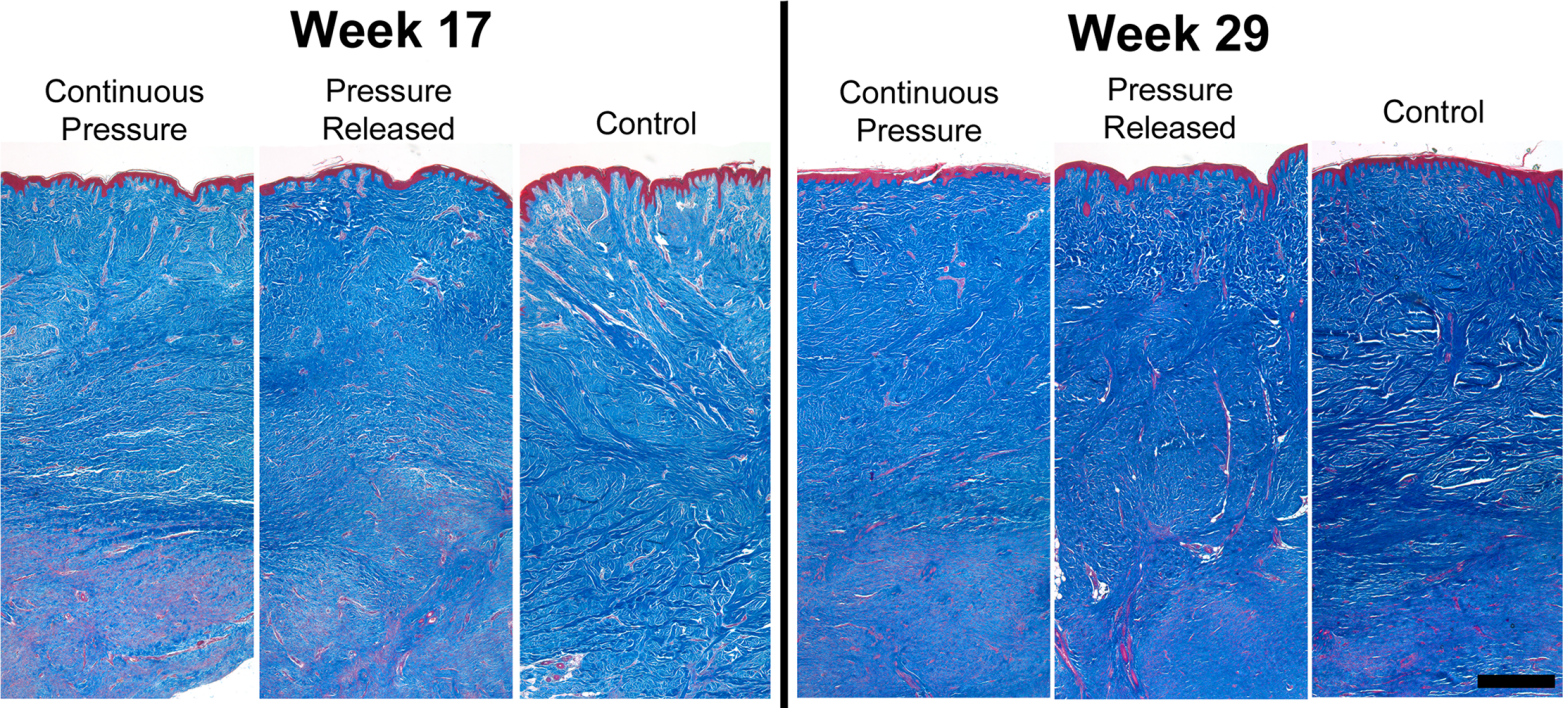
**Representative images from trichrome stained histological sections prior to pressure release (left) and at the final time point (right).** Pressure garment therapy resulted in less dense matrix deposition, indicated by a lighter blue stain. Over time, collagen deposition increased in all groups, but the density was greater in the pressure released and control groups at week 29. Scale bar = 750 μm.

Trichrome stained histological sections showed thicker, denser collagen matrix (blue stain) in control scars at week 17 compared to scars that were undergoing PGT (Fig 7). Pressure treated scars showed a larger portion of the dermis that contained a greater amount of cell cytoplasm (red stain) compared to control scars. By week 29, the collagen content and density of all treatment groups increased, indicated by the darker blue stain, though scars in the released and control groups appeared to have a denser matrix than scars that received continuous pressure.

**Fig 8:**
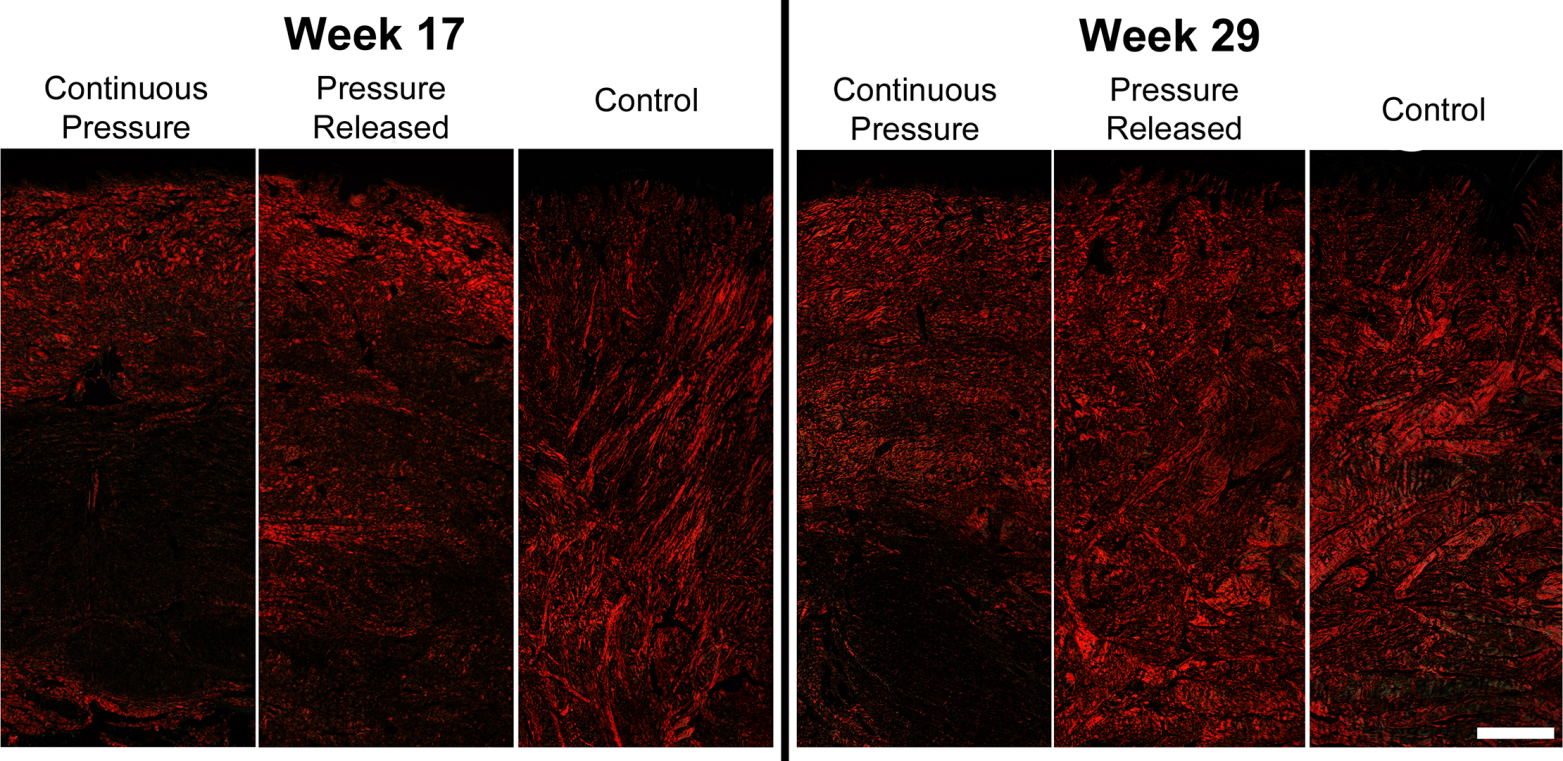
**Representative images of Picrosirius Red stained histological sections prior to pressure released (left) and at the final time point (right).** Pressure treated groups contained thinner collagen fibers oriented parallel to the surface of the scars. Cessation of pressure resulted in the formation of thick collagen bundles oriented perpendicular to the epidermis. Scale bar = 500 μm.

**Fig 9:**
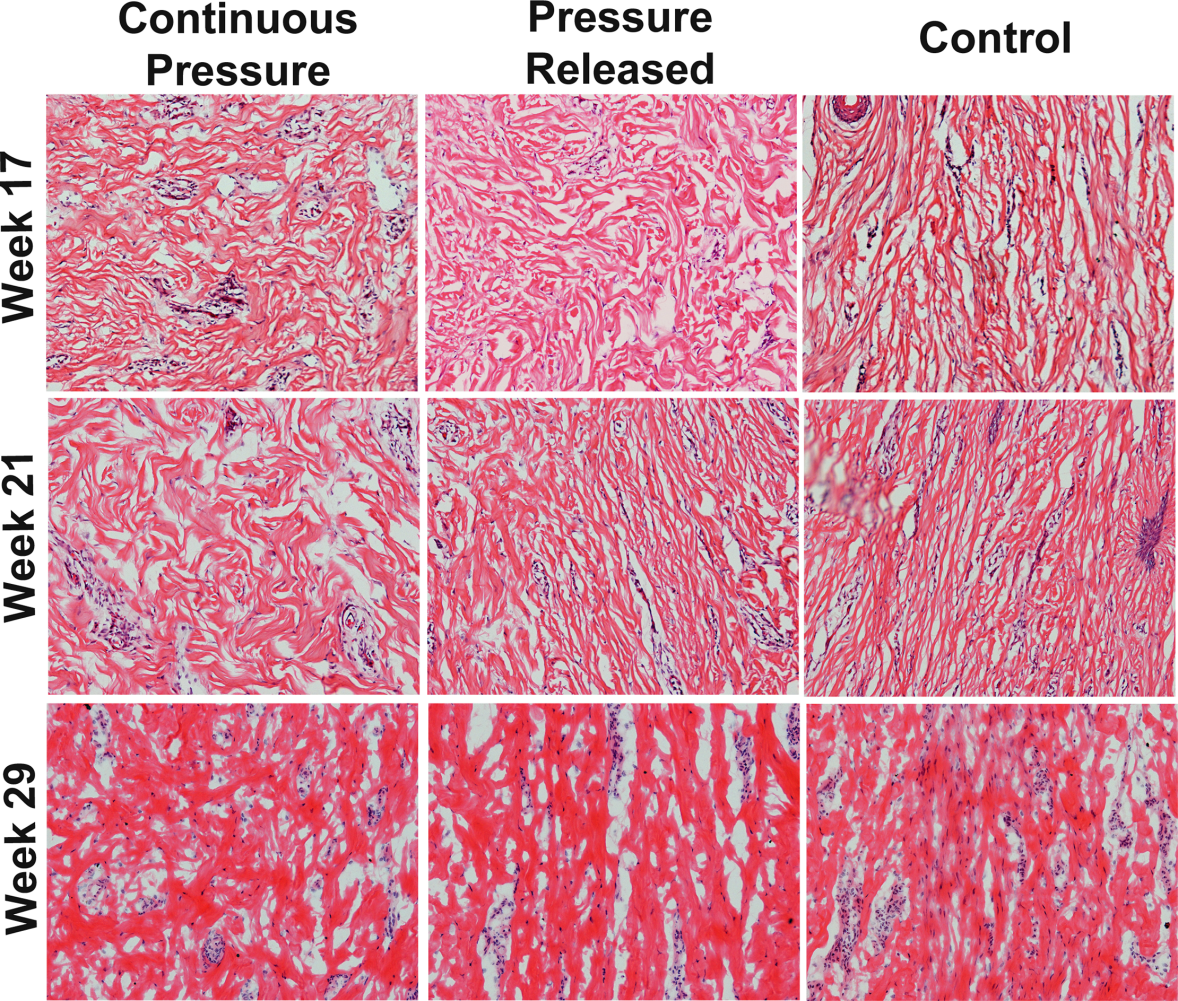
Histological sections of scars cut *en face* showing extracellular matrix organization as a function of treatment and time. Collagen fibers in the groups receiving pressure garment therapy (PGT) were oriented in two primary directions whereas controls were oriented parallel to the circumference of the pig (the vertical axis in each image). After cessation of PGT, collagen fibers within the pressure released group became more uniformly oriented in one direction. This increase in alignment continued until the end of the experiment (Week 29).

Picrosirius Red staining at week 17 revealed that the collagen fibers in the control group tended to be thicker, compared to the thinner collagen fibers in the pressure treated groups that were oriented parallel to the surface of the scars (Fig 8). At week 29, fibers in the continuous pressure group continued to be thin and parallel, while the pressure released group became much thicker. Fiber thickness in the control group also increased from weeks 17 to 29. *En face* sectioning of the scar showed that scars treated with PGT contained fibers that were organized in a cross-hatch pattern whereas the collagen within the control scars was highly aligned in the dorsal-ventral direction (Fig 9). When PGT was halted in the pressure released group, a substantial increase in collagen alignment was observed within 4 weeks of release with fibers largely oriented in the dorsal-ventral orientation as observed in the control group. At week 29, alignment in the released and control groups was still observed; however it was obscured slightly by the increase in fiber thickness (Fig 9).

## Discussion

During the maturation of hypertrophic scars, collagen synthesis is elevated compared to normal skin and normal scars, and can remain elevated for 2–3 years after injury[16]. During this time, there is an increase in collagen fibril thickness[17–19] and number of cross links[18]. Picrosirius Red staining revealed a marked increase in the thickness of collagen fibrils in the control scars that occurred between weeks 17 and 29, indicating that the scars were not mature and still undergoing high rates of collagen synthesis. When pressure garments are removed, there is an increase in the biomechanical forces applied to the scars due to the tension of the surrounding skin. Prior studies have shown that applying uniaxial tension to reconstituted collagen *in vitro* results in decreased degradation of the collagen fibrils compared to relaxed control samples when exposed to either collagenase[20] or matrix metalloproteinase-8[21]. The collagen matrix in both studies was acellular, suggesting that a physical change in collagen morphology, driven by the mechanical environment of the dermis, is capable of altering collagen accumulation within the scar. We hypothesize that when PGT is halted and the compressive forces removed, tension is quickly re-established within the scar, altering the balance between collagen degradation and deposition.

Additionally, it has been reported that fibroblasts on aligned matrices up-regulate collagen synthesis compared to randomly aligned matrices[22,23]. As the release of pressure resulted in increased collagen alignment in the current study, it is likely that fibroblasts within this group produced more collagen (as evidenced by the increase in scar thickness from week 17 to 29). This increase in collagen deposition coupled with a greater resistance to degradation imparted by the mechanical tension on the scar may accelerate the scar thickening and contraction process. It is possible that maintaining pressure until the scar is fully matured, and collagen synthesis has begun to decrease, may prevent this disparity between synthesis and degradation, and allow the benefits of PGT to be preserved.

The pressure released group in the current study showed rapid scar contraction after pressure removal; within 4 weeks the scars had significantly decreased area compared to the continuous pressure group. Wound contraction is often attributed, in part, to the differentiation of fibroblasts into myofibroblasts[24]. An increase in myofibroblast differentiation, indicated by expression of α-smooth muscle actin (α-SMA)[25], has been correlated with increased wound contraction in porcine burn wounds[26]. Multiple studies have shown that fibroblasts cultured *in vitro* differentiate into myofibroblasts in response to increased stress[27–29], with significant increases in α-SMA observed in as little as 6 hours following loading[27]. Conversely, a study conducted on human hypertrophic scars undergoing PGT found a significant decrease in α-SMA after 3 months of PGT compared to scars that were not treated with pressure[30]. Removing pressure on the immature scars in the current study could have resulted in an increase in strain and subsequent differentiation of fibroblasts, resulting in the rapid contraction exhibited in this study.

## Conclusion

PGT therapy resulted in scars that were smoother, less raised, and less contracted after four months compared to control scars. However, removing pressure prior to scar maturation resulted in a rapid increase in scar contraction and significant scar thickening resulting in scars that were not statistically different than scars that had never been treated with PGT. Scars that received uninterrupted pressure continued to maintain observed benefits. These changes in scar properties after pressure cessation support the need to continue PGT for greater lengths of time. Additional studies will be needed to determine the duration of PGT required to achieve maximum benefit without regression after pressure removal.

## Supporting information

S1 Dataset Raw data for scar area, roughness, biomechanics and scar height. (XLSX)
